# Impact of personality hardiness on anxiety dynamics during the COVID-19 outbreak in russia

**DOI:** 10.1192/j.eurpsy.2021.763

**Published:** 2021-08-13

**Authors:** D. Dovbysh, V. Epishin, A. Salikhova, N. Bogacheva, M. Bogdanova, M. Kiseleva

**Affiliations:** Pedagogy And Medical Psychology, Federal State Autonomous Educational Institution of Higher Education I.M. Sechenov First Moscow State Medical University of the Ministry of Health of the Russian Federation (Sechenov University), Moscow, Russian Federation

**Keywords:** Anxiety, COVID-19, hardiness, Depression

## Abstract

**Introduction:**

Hardiness is a set of attitudes, providing courage and motivation to cope with stress (Maddi, 2006). The COVID-19 outbreak and the response to it caused exceptional stress and drastically changed the everyday routine, endangering many people`s psychological well-being and mobilizing coping resources.

**Objectives:**

The study aimed to determine whether hardiness provided coping resources to deal with COVID-19 outbreak-related stressors.

**Methods:**

949 participants from Russia (ages 18-66) voluntarily completed online questionnaires: BAI; BDI; SCL-90-R; Personal Views Survey III during the early COVID-19 restrictions (24 March - 15 May). Subsamples from four time periods were compared using ANOVA. The first dataset was collected before the official restrictions’ introduction (n=88). The second subsample was gathered during the “days off” week (n=262). The third period started with the “days off” extension and ended with the strict self-isolation announcement (n=296). The fourth dataset was gathered during self-isolation (n=303). General linear models (GLM) were used to determine the effect of variables on anxiety, depression, and general symptomatic index (GSI).

**Results:**

Hardiness, anxiety, depression, and GSI differed significantly between the time-periods (F=4.899, p<0.01; F=3.173, p<0.05; F=8.096, p<0.01; F=3.244, p<0.022 ; F=4.899, p<0.01 respectively). GLMs showed gender, chronic diseases, self-assessed fears, and hardiness contribution to anxiety, depression, and GSI. Hardiness had the biggest effect on all models. Anxiety was additionally influenced by the time factor, which also interacted with hardiness (see Figure 1). With lower hardiness, higher anxiety arose over time.

**Figure:**
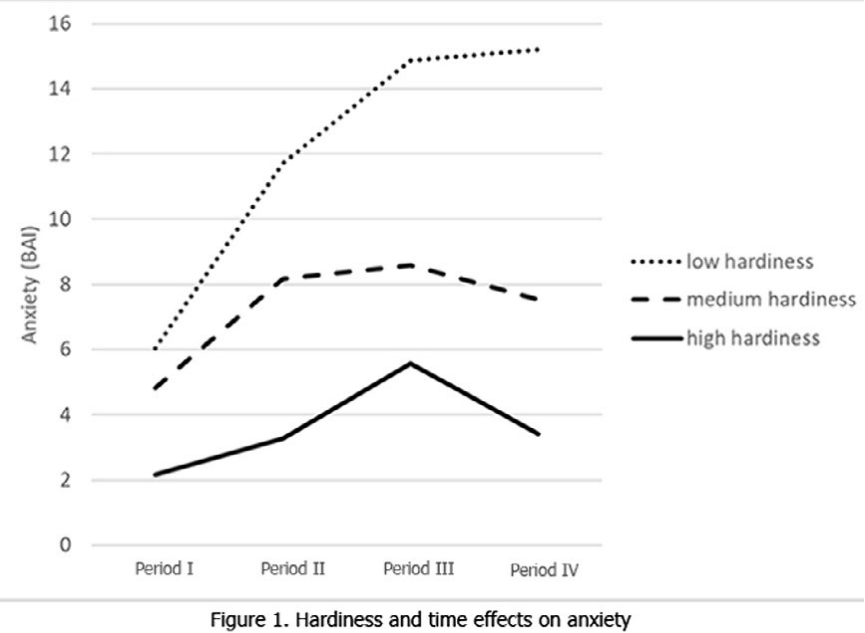

**Conclusions:**

Hardiness notably contributes to personal adaptation during the COVID-19 outbreak-related restrictions.

